# Homelessness and housing assistance among persons with HIV, and associations with HIV care and viral suppression, New York City 2018

**DOI:** 10.1371/journal.pone.0285765

**Published:** 2023-05-12

**Authors:** Ellen Weiss Wiewel, Yaoyu Zhong, Qiang Xia, Christopher M. Beattie, Paul A. Brown, Pam X. Farquhar, John F. Rojas

**Affiliations:** 1 New York City Department of Health and Mental Hygiene, Long Island City, NY, United States of America; 2 New York City Department of Social Services, New York, NY, United States of America; University of Arkansas for Medical Sciences, UNITED STATES

## Abstract

**Objectives:**

To measure housing assistance and homelessness among persons living with HIV (PLWH) and their association with health.

**Methods:**

Exposure categories were: experiencing homelessness (per emergency shelter use or self-report), receiving housing assistance (per housing subsidy) without homelessness, or neither homelessness nor receiving housing assistance. Outcomes were: engagement (≥1 visit) and retention (≥2 visits ≥90 days apart) in HIV-related medical care and one-time (latest viral load) and durable (≥1 viral load test, all suppressed) HIV viral suppression (<200 copies/mL). Among PLWH in New York City (NYC), we calculated and conducted modified Poisson regressions of the four outcomes according to exposure category.

**Results:**

During 2018, 45% of NYC’s 84,053 PLWH received housing assistance, and 8% experienced homelessness. Relative to homelessness, receipt of assistance without homelessness was associated with 3–7% higher adjusted relative risk (ARR) of engagement and retention in care and 31–64% higher ARR of one-time and durable viral suppression. Relative to not receiving assistance, receipt of assistance without homelessness was associated with 6–18% higher ARR of care and 2–5% lower ARR of viral suppression.

**Conclusions:**

Programs promoting housing stability may support HIV care and viral suppression, particularly if preventing homelessness. These may help improve HIV care and suppression rates.

## Introduction

HIV diagnosis and prevalence rates are disproportionately high among persons with low income or unstable housing [1]. Homeless and unstably housed persons living with HIV (PLWH) generally are less likely than stably housed PLWH to be engaged in HIV-related medical care and to achieve and maintain HIV viral suppression [2, 3]. Engagement in care allows for medical monitoring and counseling, and the prescription of HIV medication, called antiretroviral therapy (ART). HIV viral suppression is a medical status that indicates that the individual’s infection is controlled at the moment and cannot be transmitted sexually [4, 5]; suppression is typically achieved and maintained by taking ART daily.

It has been said that “housing is healthcare,” [6] meaning that safe, stable housing contributes to wellbeing and helps people manage illness and infection. United States federal and state plans responding to the HIV epidemic typically reflect this value. For example, the US National HIV/AIDS Strategy set a goal of reducing the percentage of persons in HIV medical care who experience homelessness to no more than 5 percent, from a 2010 baseline of 7.7%; that figure unfortunately stood at 9% in 2014 [7]. The New York State “Ending the Epidemic” blueprint recommends ensuring access to stable housing for persons living with or vulnerable to HIV [8].

In New York City (NYC), numerous programs operate to alleviate housing challenges among PLWH. Some of these programs have been shown to improve rates of care engagement and retention and sometimes viral suppression, as well [9–11]. However, the total numbers of PLWH in NYC receiving housing assistance or experiencing homelessness and seeking emergency shelter, and the proportion of all PLWH that they comprise, have not been calculated recently [1]. Further, how health outcomes among PLWH differ according to receipt of housing services in NYC has not been assessed comprehensively. Are care outcomes as good among PLWH receiving housing assistance as those not, or better, and worse among those who experienced homelessness? And what might this suggest about the capacity of economic policies that alleviate poverty and promote housing stability to restore HIV care engagement and viral suppression, which declined among all PLWH during the COVID-19 pandemic? [12]. To address these gaps in knowledge, and to inform strategies to restore and further increase care and suppression rates, we described demographic characteristics, HIV-related medical care, and HIV viral suppression among adult PLWH in NYC, among those experiencing homelessness, receiving housing assistance without homelessness, or neither homeless nor receiving housing assistance. We also tested whether these three groups had different rates of engagement and retention in HIV-related medical care, or of one-time or durable HIV viral suppression, after controlling for other characteristics.

## Methods

### Data sources

We used multiple linked data sources reported to the NYC Department of Health and Mental Hygiene by April 2019 for information about HIV and receipt of housing services during 2018. These included data on PLWH receiving housing services through: (a) the NYC Human Resources Administration’s HIV/AIDS Services Administration (HASA), through which most low-income PLWH in NYC are eligible for housing assistance, and which is funded by City, State, and Federal programs; (b) the HIV housing units funded by a joint NYC/New York State supportive housing program called New York / New York (NY/NY) III; (c) the NYC portfolio of housing contracts funded by the federal Housing Opportunities for Persons with AIDS (HOPWA) grant of the U.S. Department of Housing and Urban Development; (d) the NYC portfolio of housing contracts funded by the federal Ryan White Part A grant of the U.S Human Resources and Services Administration; and (e) the NYC Department of Homeless Services (DHS) shelter system.

Data from these five housing programs were linked to the NYC HIV surveillance registry, which provided data related to HIV diagnosis, vital status, place of residence, demographics, and laboratory test results, for housing consumers as well as all other persons diagnosed with HIV and reported in NYC.

### Population

The analysis included the estimated population of all persons 18+ years old who were diagnosed and living with HIV at the end of 2018 and presumed to be living in NYC. The total number and traits of this estimated population were characterized using a previously described weighting method [13, 14].

Briefly, the NYC HIV laboratory data reporting system was treated as a special annual population-based survey, lasting from January 1 to December 31 each year. Every PLWH in NYC had a non-zero, unequal probability of participating in this annual survey, i.e., receiving HIV care in NYC in each calendar year. People with regular care were more likely to be included in the survey than people with sporadic care. For this analysis, people who received at least one CD4 or viral load test in NYC in 2018 were considered survey participants and each participant was given a weight that was the inverse of the probability of receiving at least one CD4 or viral load test in NYC in 2018. The probability was calculated based on the time interval between the last care visit prior to 2018, or the date of diagnosis if no care visits prior to 2018, and the first care visit in 2018. If a person’s time interval was ≤1 year, meaning that they were in regular care and would participate in the survey with a probability of 100%, they would receive a weight of 1 and represent only themself; if a person’s time interval was greater than 1 year, meaning that they could have been out of care in 2018 and not participated in the survey, they would receive a weight equal to the time interval in years and represent themself and the weight minus 1 number of out-of-care people. For example, a person in care in June 2018 whose last care visit was exactly three years prior in June 2015 received a weight of three, and the person represented themself plus two persons out of care in 2018.

Using this weighing method, we were able to estimate the number of out-of-care persons and their traits. The total population of PLWH was estimated as the actual persons in care plus the estimated persons out of care. We then subtracted from the weighted estimates the persons known to have experienced homelessness or received housing assistance, to obtain estimates of PLWH neither experiencing homelessness nor receiving housing assistance.

### Exposures

Classifying persons according to receipt of housing assistance and homelessness, we defined three exposure groups for analyses: PLWH experiencing homelessness, PLWH receiving housing assistance and not experiencing homelessness, and PLWH neither receiving housing assistance nor experiencing homelessness. Housing assistance was defined as receiving any housing subsidy or emergency shelter (including homeless shelter) during the year, from HASA, NY/NY III, HOPWA, Ryan White, and/or DHS. Homelessness was defined as using a DHS shelter or HASA emergency single room occupancy unit (SRO) or reporting homelessness (per housing program records; includes living in a place not meant for human habitation such as a street, couch-surfing or temporarily staying with others, or staying in an emergency shelter or SRO hotel) at any point during the year.

This definition of homelessness is in keeping with what the Housing and Urban Development categorizes as “Category 1” homelessness, or “literally homeless” [15]. In addition to street homelessness, this includes individuals or families residing in a “publicly or privately operated shelter designated to provide temporary living arrangements,” including, “hotels and motels paid for by charitable organizations or by federal, state and local government programs.” Residence in HASA transitional supportive housing was not included in the definition of homelessness because residents receive supportive services including assistance in securing permanent housing, which shelters, SROs and other temporary housing typically do not offer.

For this analysis, persons experiencing homelessness were defined as a subset of all persons receiving housing assistance, as individuals who did not interact with the government-affiliated housing services system could not be identified as homeless.

Key measures of interest were the proportion of all PLWH who received housing assistance; and the proportion of all PLWH, PLWH in care, and PLWH receiving housing assistance who experienced homelessness.

### Outcomes

Outcomes were engagement in HIV-related medical care (at least one viral load [VL] or CD4 in 2018), retention in care (at least 2 visits at least 90 days apart in 2018), viral suppression (last VL in 2018 was <200 copies/mL), and durable suppression (all VL in 2018 were <200 copies/mL).

### Statistical analysis

To compare distributions of covariates and care outcomes across groups by housing assistance and homelessness, we used Chi-square tests of independence. Because risk ratio (RR) is the parameter of the interest of our study and log-binominal models failed to converge, we ran modified Poisson regressions with the outcomes being each of the four care outcomes, to obtain RRs [16, 17]. Although Poisson regression is not often used for binary outcomes, it is appropriate when using a robust error variance procedure (known as sandwich estimation) [18]. A simulation study showed that the modified Poisson regression produced very reliable estimation for RR even with total sample sizes as small as 100 and had no difficulty with converging, compared with the binomial regression [16]. We controlled for age (18–24 years old, 25–34, 35–44, 45–54, 55–64, 65+), current gender identity (man, woman), transmission risk (men who have sex with men and do not inject drugs [MSM], persons who inject drugs, heterosexuals who do not inject drugs, all other persons), race/ethnicity (Black, Latino/a, White, all other persons), and HIV diagnosis year (pre-1990, 1991–1995, 1996–2000, 2001–2005, 2006–2010, 2011–2015, 2016–2018). These categories were broader than the more-granular distributions of gender and risk in initial frequencies. We ran one adjusted model twice, facilitating comparisons between all three exposure groups by having two different reference groups: PLWH experiencing homelessness (Model 1a) and PLWH neither receiving housing assistance nor experiencing homelessness (Model 1b).

### Ethical review and data availability

This analysis evaluates the extent of enrollment in housing programs among persons with HIV, and associated health outcomes. The study’s methods and client confidentiality and privacy were reviewed and determined to not require institutional review board (IRB) review. Individual-level data may not be made publicly available.

## Results

During 2018, there were 84,053 PLWH in NYC, among whom 31,528 (38%) were found to be receiving housing assistance without homelessness (Table 1). There were 6,567 PLWH in NYC experiencing homelessness that year, accounting for 8% of all PLWH, 6% of PLWH in care, and 17% of persons receiving some form of housing assistance, i.e., either a housing subsidy or emergency shelter. The remaining 55% of PLWH were that year neither receiving housing assistance nor experiencing homelessness.

**Table 1:** Demographics among persons living with HIV, by housing assistance and homelessness, New York City 2018.

	Total	PLWH receiving housing assistance										PLWH neither receiving housing assistance nor experiencing homelessness		
		Subtotal			PLWH experiencing homelessness			PLWH receiving housing assistance and not experiencing homelessness							
	N	N	Column	Row	N	Column	Row	N		Column		N		Column	
***Total***	*84*,*053*	*38*,*095*	*100%*	*45%*	*6*,*567*	*100%*	*8%*	*31*,*528*		*100%*		*45*,*958*		*100%*	
**Gender**															
Cisgender male	59,217	25,191	66%	43%	4,717	72%	8%	20,474		65%		34,026		74%	
Cisgender female	23,176	11,606	30%	50%	1,451	22%	6%	10,155		32%		11,570		25%	
Transgender woman	1,631	1,279	3%	78%	394	6%	24%	885		3%		352		1%	
Transgender man	28	19	0%	68%	5	0%	18%	14		0%		9		0%	
**Age in 2018**															
18–24	1,948	951	2%	49%	280	4%	14%	671		2%		997		2%	
25–34	12,574	6,271	16%	50%	1,642	25%	13%	4,629		15%		6,302		14%	
35–44	14,246	6,696	18%	47%	1,374	21%	10%	5,322		17%		7,550		16%	
45–54	22,494	10,473	27%	47%	1,723	26%	8%	8,750		28%		12,021		26%	
55–64	22,921	10,266	27%	45%	1,288	20%	6%	8,978		28%		12,655		28%	
65+	9,870	3,438	9%	35%	260	4%	3%	3,178		10%		6,432		14%	
**Race/ethnicity**															
Black	38,260	19,904	52%	52%	3,880	59%	10%	16,024		51%		18,356		40%	
Latino/Hispanic	28,463	14,225	37%	50%	2,137	33%	8%	12,088		38%		14,238		31%	
White	14,691	3,268	9%	22%	447	7%	3%	2,821		9%		11,423		25%	
Another race or unknown	2,638	698	2%	26%	103	2%	4%	595		2%		1,940		4%	
**HIV transmission risk category**															
Men who have sex with men (MSM)	35,246	13,444	35%	38%	2,240	34%	6%	11,204		36%		21,802		47%	
Persons who inject drugs and are not MSM	8,420	5,625	15%	67%	1,107	17%	13%	4,518		14%		2,795		6%	
MSM who inject drugs	2,241	1,446	4%	65%	419	6%	19%	1,027		3%		795		2%	
Heterosexual contact	18,363	8,723	23%	48%	1,279	19%	7%	7,444		24%		9,640		21%	
Transgender people with sexual contact	1,429	1,098	3%	77%	329	5%	23%	769		2%		331		1%	
Perinatal	1,533	939	2%	61%	155	2%	10%	784		2%		594		1%	
Other	122	55	0%	45%	2	0%	2%	53		0%		67		0%	
Unknown	16,700	6,765	18%	41%	1,036	16%	6%	5,729		18%		9,935		22%	
**Year of HIV diagnosis**															
Pre-1990	5,563	2,839	7%	51%	450	7%	8%	2,389		8%		2,724		6%	
1991–1995	10,172	5,267	14%	52%	755	11%	7%	4,512		14%		4,905		11%	
1996–2000	17,685	8,297	22%	47%	1,027	16%	6%	7,270		23%		9,388		20%	
2001–2005	16,397	7,740	20%	47%	1,121	17%	7%	6,619		21%		8,657		19%	
2006–2010	14,783	6,698	18%	45%	1,266	19%	9%	5,432		17%		8,085		18%	
2011–2015	12,868	5,173	14%	40%	1,259	19%	10%	3,914		12%		7,695		17%	
2016–2018	6,586	2,081	5%	32%	689	10%	10%	1,392	4%		4,505		10%	

PLWH receiving housing assistance and not experiencing homelessness were more likely than PLWH experiencing homelessness or PLWH not receiving housing assistance to be cisgender women (32%, compared with 22% and 25%, respectively) and diagnosed by the year 2000 (45%, compared with 33% and 37%, respectively) (Table 1; all *p*<0.001). PLWH receiving housing assistance, whether experiencing homelessness or not, were more likely than PLWH not receiving housing assistance to be Black or Latino (92% and 89%, respectively, compared with 71%; *p*<0.001) and persons who inject drugs, whether MSM or not (23% and 17%, respectively, compared with 8%; *p*<0.001). Compared with PLWH who had not been homeless or not receiving housing assistance, PLWH experiencing homelessness were more likely to be under 25, transgender, Black, persons who inject drugs, whether MSM or not, and diagnosed with HIV after 2010 (all *p*<0.001).

Of the 38,095 persons receiving housing assistance, 95% were engaged in care, 81% retained in care, 78% virally suppressed, and 64% durably suppressed (Table 2). Care and suppression rates were lowest among the subset who experienced homelessness: 92% were engaged in care (compared with 96% among those receiving housing assistance and not experiencing homelessness, for example), 74% retained in care (compared with 83%), 62% virally suppressed (compared with 82%), and 39% durably suppressed (compared with 69%; all *p*<0.001).

**Table 2:** Medical care and viral suppression among persons living with HIV (PLWH), by housing assistance and homelessness, New York City 2018.

	Total	Engaged in care		Retained in care		Virally suppressed		Durably suppressed	
		N	Row	N	Row	N	Row	N	Row
Total	*84*,*053*	*78*,*413*	*93%*	*63*,*192*	*75%*	*68*,*983*	*82%*	*58*,*993*	*70%*
PLWH receiving housing assistance, total	*38*,*095*	36,327	95%	30,895	81%	29,714	78%	24,307	64%
PLWH experiencing homelessness	*6*,*567*	6,039	92%	4,843	74%	3,966	60%	2,593	39%
PLWH receiving housing assistance and not experiencing homelessness	*31*,*528*	30,288	96%	26,052	83%	25,748	82%	21,714	69%
PLWH neither receiving housing assistance nor experiencing homelessness	*45*,*958*	42,086	92%	32,297	70%	39,269	85%	34,686	75%

In the multivariable model, compared with PLWH experiencing homelessness, persons receiving housing assistance and not experiencing homelessness were more likely to be engaged (adjusted risk ratio [ARR] = 1.03; 95% confidence interval: 1.02, 1.04) and retained in care (1.07 [1.06, 1.09]), and more likely to be virally suppressed both at last viral load (1.31 [1.28, 1.33]) and durably (1.64 [1.59, 1.69]; Table 3). PLWH neither receiving housing assistance nor experiencing homelessness had similar engagement as PLWH experiencing homelessness and were less likely to be retained (ARR = 0.91[0.89, 0.92]) but more likely to be virally suppressed both at last viral load (1.33 [1.31, 1.36]) and durably (1.73 [1.68, 1.79]) (model 1a). With PLWH neither receiving housing assistance nor experiencing homelessness as the reference group (model 1b), persons receiving housing assistance and not experiencing homelessness were more likely to be engaged (1.06 [1.05, 1.07]) and retained in care (1.18 [1.17, 1.19]), and less likely to be virally suppressed both at last viral load (0.98 [0.97, 0.99]) and durably (0.95 [0.94, 0.96]).

**Table 3:** The association of housing assistance and homelessness with HIV care and viral suppression among persons living with HIV (PLWH), New York City 2018.

	Engaged in care		Retained in care		Virally suppressed		Durably suppressed	
	RR (95% CI)	ARR (95% CI)	RR (95% CI)	ARR (95% CI)	RR (95% CI)	ARR (95% CI)	RR (95% CI)	ARR (95% CI)
**Model 1a**								
PLWH experiencing homelessness (ref)	1.00	1.00	1.00	1.00	1.00	1.00	1.00	1.00
PLWH receiving housing assistance and not experiencing homelessness	1.04 (1.04–1.05)	1.03 (1.02–1.04)	1.12 (1.10–1.14)	1.07 (1.06–1.09)	1.35 (1.33–1.38)	1.31 (1.28–1.33)	1.74 (1.69–1.80)	1.64 (1.59–1.69)
PLWH neither receiving housing assistance nor experiencing homelessness	1.00 (0.99–1.00)	0.97 (0.96–0.98)	0.95 (0.94–0.97)	0.91 (0.89–0.92)	1.41 (1.39–1.44)	1.33 (1.31–1.36)	1.91 (1.85–1.97)	1.73 (1.68–1.79)
**Model 1b**								
PLWH experiencing homelessness	1.00 (1.00–1.01)	1.03 (1.02–1.04)	1.05 (1.03–1.07)	1.10 (1.08–1.12)	0.71 (0.69–0.72)	0.75 (0.74–0.77)	0.52 (0.51–0.54)	0.58 (0.56–0.60)
PLWH receiving housing assistance and not experiencing homelessness	1.05 (1.04–1.05)	1.06 (1.05–1.07)	1.18 (1.17–1.19)	1.18 (1.17–1.19)	0.96 (0.95–0.96)	0.98 (0.97–0.99)	0.91 (0.90–0.92)	0.95 (0.94–0.96)
PLWH neither receiving housing assistance nor experiencing homelessness(ref)	1.00	1.00	1.00	1.00	1.00	1.00	1.00	1.00

The multivariable model was adjusted for age, current gender identity, transmission risk category, race/ethnicity, and HIV diagnosis year.

## Discussion

We found that housing needs are widespread among NYC PLWH, and PLWH experiencing homelessness have worse HIV care and suppression outcomes. Tens of thousands of NYC PLWH have housing needs, and nearly all of them are receiving some form of housing assistance to meet those needs, mostly as long-term rent subsidies. (A minority of PLWH were in the city’s homeless shelters.) With 8% of NYC PLWH in care experiencing homelessness, we are not yet meeting the 2020 NHAS target of < = 5% [7], nor the 2015 NYS recommendation to “ensure access to stable housing” for PLWH [8]. To the extent that we are close to these targets, we have accomplished this by providing and bolstering housing and support services for PLWH via federal programs like Ryan White and HOPWA and local initiatives like HASA and other affordable housing.

Compared with PLWH experiencing homelessness, PLWH receiving housing assistance and not experiencing homelessness had better outcomes across all four care and suppression outcomes. Compared with PLWH experiencing homelessness, or PLWH receiving housing assistance and not experiencing homelessness, PLWH neither receiving housing assistance nor experiencing homelessness had only better suppression outcomes, but not better engagement or retention in care. These findings are consistent with prior knowledge that housing services that prevent homelessness among PLWH may support engagement and retention in care [3, 10, 19–21]. Indeed, these programs, including Ryan White and HOPWA, emphasize connection to and retention in care as part of their services. People not receiving housing assistance, who are also taking their ART regularly and achieving viral suppression, may have less frequent visits, because their providers do not need to monitor them as frequently, and they do not have a social services program promoting more frequent visits. This potentially accounts for the higher suppression rates, without higher engagement or retention, among those not receiving assistance.

Our results echo findings from another cross-sectional study indicating that PLWH receiving housing assistance were not more likely than those not receiving housing assistance to achieve viral suppression [10]. However, more recent analyses have demonstrated an effect of housing assistance on both care and viral suppression [2, 9, 11]. It may be partly because some persons who received housing assistance may have done so for only a short time by the end of the year, possibly before they were able to achieve viral suppression [9]. Additionally, PLWH receiving housing assistance have low incomes, may live in neighborhoods with higher poverty rates, are more likely to be Black and Hispanic and thus more subject to racism and discrimination, and are more likely to have mental illness or substance use disorder than PLWH not receiving housing assistance, so they may experience worse housing stability and housing quality even if receiving housing assistance; this possibly complicates adherence to their ART regimens [2]. Those numerous challenges among PLWH receiving housing assistance notwithstanding, disparities in care and suppression outcomes by housing group were starkest when comparing the outcome of durable suppression between PLWH experiencing homelessness–who were least likely to be durably suppressed–and PLWH either not receiving housing assistance or receiving it and not experiencing homelessness. This finding underscores the importance of housing assistance itself and housing programs’ help with connection to and retention in care.

NYC has taken proactive steps to improve health and housing stability among PLWH. NYC is an integral partner of the New York State’s Blueprint to End the AIDS Epidemic [8]. In 2016, NYC expanded its eligibility for the HASA program and its rental subsidies: it now includes all low-income NYC residents living with HIV, regardless of stage of disease progression [22]. By 2018, this newly eligible population of persons living with HIV only (non-AIDS) comprised 6,000 of 38,000 HASA consumers. In addition, DHS, HASA, and DOHMH collaborate to identify and conduct outreach with homeless PLWH residing in DHS shelters with the intent of connecting them to medical care, more stable housing, and benefits. This effort individually engaged 125 PLWH who lived in DHS homeless shelters in the fourth quarter of 2018 (unpublished data, NYC DOHMH). NYC has also expanded its affordable housing for low-income persons generally, including those in need of supportive housing [23].

Some of the same structural inequalities that contribute to HIV acquisition also contribute to housing instability. This was apparent in findings such as those by race and ethnicity: Black and Hispanic persons comprised a greater proportion of PLWH receiving housing assistance, including persons experiencing homelessness, than PLWH not receiving housing assistance (89% vs. 71%). We did not have data on income.

### Limitations

We were not able to identify in our analysis PLWH with serious housing needs who were *not* receiving housing assistance or homelessness services through government-affiliated programs, such as persons living on the street or in transit facilities or couch-surfing. Most PLWH experiencing homelessness in NYC are not living on the street, but rather, reside in HASA or DHS housing. Nevertheless, it was estimated that NYC had 3,675 persons living on the street or in transit facilities in January 2018 [24]. HIV prevalence among this population is not known, but if it were the same as that among persons living in DHS shelters in 2018 (0.62%; unpublished data, NYC DOHMH and DHS), about 23 persons living on the street or in transit facilities could be living with HIV. This is a relatively small increase to the total number of New Yorkers living with HIV who have housing needs, but this population may be even less likely to be engaged in HIV care, on ART, and virally suppressed, meaning that we may have slightly overestimated rates of these outcomes in 2018 among New York’s population of PLWH with housing needs.

Non-HIV-specific housing assistance, such as Section 8 vouchers and NYCHA public housing, is also available in NYC; these two programs served over half a million New Yorkers (1 in 15) as of March 2019 [25]. Persons receiving only these types of assistance and not HIV-specific assistance would be misclassified in this analysis as not receiving assistance. Given the wide span and benefits of the city’s HIV-specific housing programs, this may be a relatively small population of PLWH and thus unlikely to affect findings.

### Strengths

These analyses had numerous strengths. We linked administrative data sources for multiple housing services in NYC, including all of the major ones for PLWH, with the records of the HIV surveillance registry. We used that link to obtain electronically reported laboratory test dates and results and determine care and suppression outcomes by receipt of housing assistance [26]. The total population of PLWH was estimated using a weighting method that accounted for care patterns and overcame limitations of surveillance such as incomplete ascertainment of population migration and out-of-jurisdiction deaths [13].

## Conclusions

Even in a US city with numerous housing programs and extensive health care resources, housing needs continue to be pervasive among PLWH. PLWH experiencing homelessness had lower rates of medical care than PLWH receiving housing assistance and not experiencing homelessness, and lower rates of viral suppression than that group and PLWH neither receiving housing assistance nor experiencing homelessness. For persons not experiencing homelessness, care engagement was actually higher among PLWH receiving housing assistance (vs. not), and suppression was only modestly lower than among those not receiving assistance. This suggests that when PLWH receive the housing assistance they need, they are better able to manage their HIV infection and, by extension, prevent transmission. Efforts to improve housing affordability, reduce structural inequalities such as poverty and racism, and support persons with mental illness and substance use disorder, may further improve HIV medical outcomes and reduce disparities. The US initiative announced in 2019, Ending the HIV Epidemic: A Plan for America [27], does not explicitly address housing, but promotion of housing stability for people living with or at risk for HIV would likely help achieve the plan’s strategies to end HIV in the US.

The COVID-19 pandemic and accompanying effects on employment have created new housing needs and vulnerabilities. Eviction moratoria, stimulus checks, enhanced unemployment benefits, and other emergency measures, including bolstered funding for HIV housing programs, have been rolled out to prevent poverty, stimulate the economy, and prevent housing instability. These measures are helping not only persons newly at risk of housing instability but also persons already enrolled in housing assistance programs, because the housing programs rarely cover the entire cost of rent: most enrollees contribute some money. Poverty mitigation and housing stabilization via continued large-scale measures will likely mitigate some of the housing-related harm of this tumultuous time. These large-scale measures are also likely to promote HIV care engagement and viral suppression for PLWH, particularly when they prevent homelessness, as long as PLWH are able to safely visit the doctor and obtain ART, such as when there are no population-wide quarantine measures nor closures of services considered non-essential.

Future research could assess the extent to which care and suppression among PLWH are negatively affected by the COVID-19 pandemic and how individual housing status and local, state, or national policies have mitigated that effect. An assessment of whether housing services attenuate racial/ethnic disparities in rates of HIV care and viral suppression among PLWH would also be valuable.
